# Transcriptomic profiles of non-embryogenic and embryogenic callus cells in a highly regenerative upland cotton line (*Gossypium hirsutum* L.)

**DOI:** 10.1186/s12861-020-00230-4

**Published:** 2020-12-02

**Authors:** Li Wen, Wei Li, Stephen Parris, Matthew West, John Lawson, Michael Smathers, Zhigang Li, Don Jones, Shuangxia Jin, Christopher A. Saski

**Affiliations:** 1 Department of Plant and Environmental Sciences, Clemson University, Clemson, SC USA; 2 Department of Food and Biology Engineering, College of Food and Chemistry Engineering, Changsha University of Science and Technology, Changsha, Hunan 410114 People’s Republic of China; 3 Cotton Incorporated, Carry, NC USA; 4 National Key Laboratory of Crop Genetic Improvement, College of Plant Science and Technology, Huazhong Agricultural University, Wuhan, People’s Republic of China

**Keywords:** *Gossypium hirsutum* L, Somatic embryogenesis, Callus, embryo

## Abstract

**Background:**

Genotype independent transformation and whole plant regeneration through somatic embryogenesis relies heavily on the intrinsic ability of a genotype to regenerate. The critical genetic architecture of non-embryogenic callus (NEC) cells and embryogenic callus (EC) cells in a highly regenerable cotton genotype is unknown.

**Results:**

In this study, gene expression profiles of a highly regenerable *Gossypium hirsutum* L. cultivar, Jin668, were analyzed at two critical developmental stages during somatic embryogenesis, non-embryogenic callus (NEC) cells and embryogenic callus (EC) cells. The rate of EC formation in Jin668 is 96%. Differential gene expression analysis revealed a total of 5333 differentially expressed genes (DEG) with 2534 genes upregulated and 2799 genes downregulated in EC. A total of 144 genes were unique to NEC cells and 174 genes were unique to EC. Clustering and enrichment analysis identified genes upregulated in EC that function as transcription factors/DNA binding, phytohormone response, oxidative reduction, and regulators of transcription; while genes categorized in methylation pathways were downregulated. Four key transcription factors were identified based on their sharp upregulation in EC tissue; *LEAFY COTYLEDON 1* (LEC1), *BABY BOOM* (BBM), *FUSCA* (FUS3) and *AGAMOUS-LIKE15* with distinguishable subgenome expression bias.

**Conclusions:**

This comparative analysis of NEC and EC transcriptomes gives new insights into the genes involved in somatic embryogenesis in cotton.

**Supplementary Information:**

The online version contains supplementary material available at 10.1186/s12861-020-00230-4.

## Background

The domain of plant transformation has enabled foundational discoveries in plant biology over the last 30 years. Plant genetic engineering has facilitated fundamental knowledge about gene function, trait genetics, and established critical linkages between the genome structure and function. Transgenic approaches have been widely used in the improvement and breeding of many crops, such as soybean, rice, and maize [1–4]. The rapid pace of genome sequencing has delivered genome maps of major domesticated crop plants and many non-model and wild species that have led to deep understandings of domestication and genetic diversity, gene function, and the development of tools for precise plant breeding [5]. However, the rate of genome characterization has far outpaced our ability to functionally profile genes and biochemical pathways in crops.

Genome editing through engineered nucleases [6] or CRISPR-Cas9 systems (spCas9-NG, base editing, xCas9, Cpf1, Cas13, Cas14) [7] are unprecedented technological breakthroughs that behold disruptive potential to precisely edit the genome of living organisms. These systems offer biologists the ability to ask precise biological questions; which has led to a new capacity in understanding gene function through targeted knockouts. Furthermore, genome editing technology has the potential to drastically reduce plant breeding cycle times and endow tailored trait genetics of elite breeding lines, which ultimately holds the potential to revolutionize commercial agriculture [5, 8]. However, major obstacles remain in deploying these frontier technologies for global crop improvement. Main bottlenecks include plant transformation and regeneration through somatic embryogenesis, and a secondary challenge is the delivery of the genome editing reagents to plant cells to produce the intended effects [5]. Agrobacterium-mediated transformation [9] and subsequent whole plant regeneration from a single somatic cell is perhaps the most historic and preferred method to produce plants homoplastic for the transgene [9]. However, this type of whole plant regeneration is generally limited to a narrow range of genotypes within a species, usually with poor agronomic traits and with low efficiency [10–12].

Somatic embryogenesis (SE) is a unique process of embryo development that involves cycles of cellular de-differentiation and reprogramming events that are controlled through signaling networks and gene expression cascades that eventually lead to embryonic cells [13]. During developmental reprograming, somatic cells de-differentiate to generate non-embryogenic cells from various explant sources, such as hypocotyls, young leaves, and immature embryos [9]. Non-embryogenic callus (NEC) cells can further differentiate to generate EC cells which function in somatic embryo development [14], which can be described by four primary stages in dicots: globular embryos, heart-shaped embryos, torpedo embryos, and cotyledon embryos [14]. Embryogenic cells are considered to be developmentally plastic and their programming of a particular developmental pathway is heavily influenced by environmental factors imposed by the tissue culture micro-environment, in addition to gene expression and regulation.

Manipulation of these programs in vitro has been achieved with some success in certain genotypes through large Design of Experiments (DOE) that determine and adjust the concentration and combination of growth regulators, such as the balance of auxin, cytokinin, and abscisic acid for each genotype [15–19]. Somatic embryogenesis is also sensitive to carbohydrate sources, inorganic salts, antibiotics, and amino acids [20–23], further adding to the complexity of the process and the difficulty of optimizing a protocol. In addition to medium components, genotype also plays a significant role in a species’ ability to regenerate, exhibiting vastly different responses across closely related genotypes [24–28].

The molecular mechanisms that orchestrate somatic embryogenesis and endow biological totipotency are becoming better understood [29]. Previous studies have shown that genes expressed during the induction of SE can be divided into three primary categories; stress-related genes, plant growth regulator (PGR) related genes, and transcription factors [30–32]. A biological stress, such as senescence or an acute stress such as abiotic/biotic factors can trigger stem cell formation through altered chromatin conformation and promiscuous expression of transcription factors [33–35]. Furthermore, these transcription factors have been shown to function in an important role on the regulation of plant differentiation [36] and development [37]. In monocots, overexpression of various plant transcription factors such as *LEAFY COTYLEDON1* [38], *WUSCHEL* [39], and *BABY BOOM* [40] have been shown to improve embryo formation and enhanced regeneration [12]. In dicots, overexpression of *BABY BOOM* to enhance regenerative capacity has been reported in tobacco [41], sweet pepper [42], and *Theobroma cacao* [43].

In upland cotton (*Gossypium hirsutum* L.), successful somatic embryogenesis was reported nearly 40 years ago [44], however mature plants were not obtained until 1983 in the genotype Coker 310 [45]. Five years later, a report that describes the optimal media formulation for the induction of somatic embryogenesis from mature and immature tissues from the genotype Coker 312 was published [46]. A follow-up study screened 38 cotton cultivars for embryogenic potential and found only Coker 312 to be responsive and concluded that embryogenic potential is genotype specific in cotton [28]. Another study substantiated that somatic embryogenesis is a heritable trait in cotton and suggested that it is polygenic because of the segregation patterns that were observed F_1_, F_2_, and BC_1_ generations of a recalcitrant by non-recalcitrant cross [47]. However, Coker lines are not well-suited for transformation studies because of their general poor agronomic qualities. In addition, Coker lines suffer from slow growth in tissue culture, a decline in vigor, and low regenerative capacity of the cultures – which includes low potency of embryogenesis, difficulty of embryo germination, a low rate of rooting, low transplant success, and a progressive loss of totipotency in culture [48]. Recently, a line with a much higher embryogenic potential and better agronomic traits was released called Jin668 [31]. This genotype is a Coker relative and was developed through successive regeneration acclimation (SRA) that involves cycling a genotype through somatic embryogenesis multiple times [31]. This continuous cycling resulted in altered DNA methylation patterns that had an effect on gene expression profiles that persisted in the germ line with a greater regeneration efficiency [31].

In this study, we analyzed and compared the transcriptional landscape of NEC and EC cells harvested from Jin668 at these critical developmental stages. We present and discuss the various classes of genes that are active during the transition from NEC to EC cells, and identify several new candidate genes that enhances our knowledge of somatic embryogenesis in upland cotton.

## Results

Somatic embryo formation efficiency of Jin668.

**Fig. 1:**
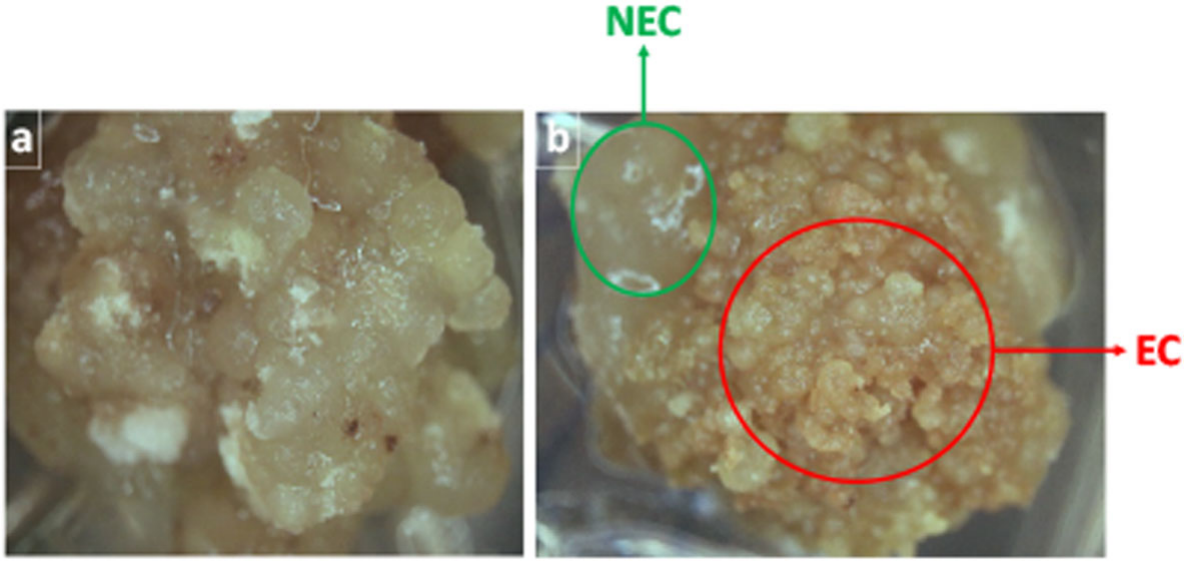
**a** 4% of the explants stay on NEC stage; **b** 96% of the explants can induced to form EC, surrounded by NEC. The examples of NEC (green circle) and EC (red circle) and the section of the callus used for RNA extraction and sequencing were circled

The embryogenic potential of Jin668 is high. NEC cells can be distinguished from EC cell by visual discrimination. NEC is characterized as fluffy, non-differentiated or polarized cells that are actively dividing, Fig. 1a. Typical EC is characterized as small cell clusters, with condensed cytoplasm, that are rapidly dividing and proliferating, Fig. 1b. After inducing callus for 45 days, 4% of the explants were found to be in the NEC stage and subsequently did not develop into embryos (Fig. 1a and Table 1). The remaining 96% of the explants were found to be induced to form characteristic embryonic callus cells (Table 1 and Fig. 1b). In all experimental replications, both NEC and EC cells could be observed in the same piece of callus (Fig. 1a and b). Therefore, those calli that consisted of both NEC and EC cells were sampled for gene expression profiling between the NEC and EC (Fig. 1b).

**Table 1:** Callus induction of cotton hypocotyls explants

No. Explants	No. NEC	No. EC (efficiency rate %)
300	12	288 (96)
320	12	308 (96)
330	13	317 (96)

### Differential gene expression and GO enrichment of NEC and EC callus

**Fig. 2:**
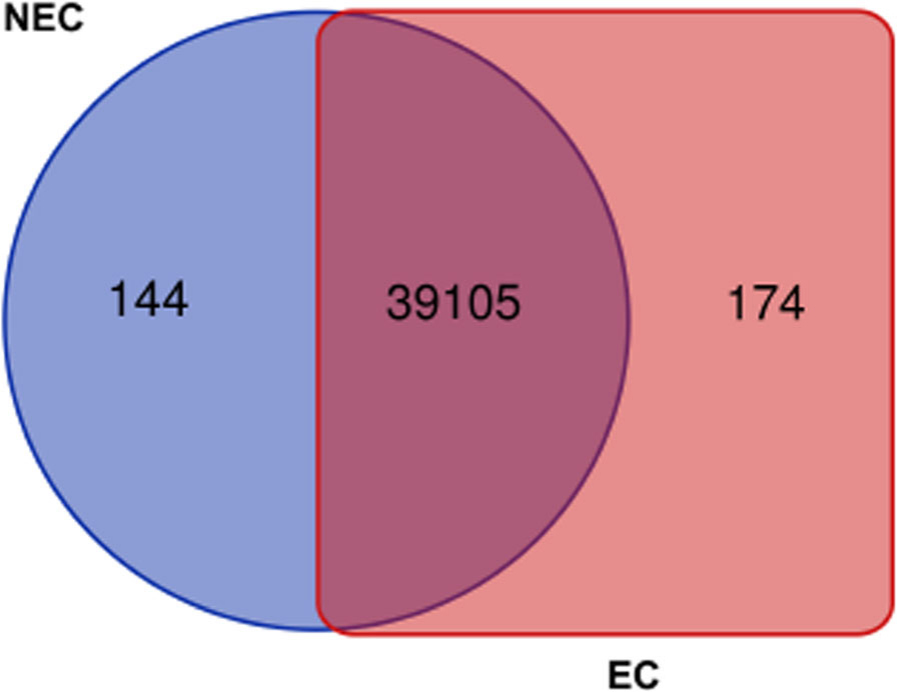
A VENN diagram of expressed genes that overlap and are unique to NEC and EC developmental stages, respectively. Genes were considered expressed when FPKM ≥ 1

**Fig. 3:**
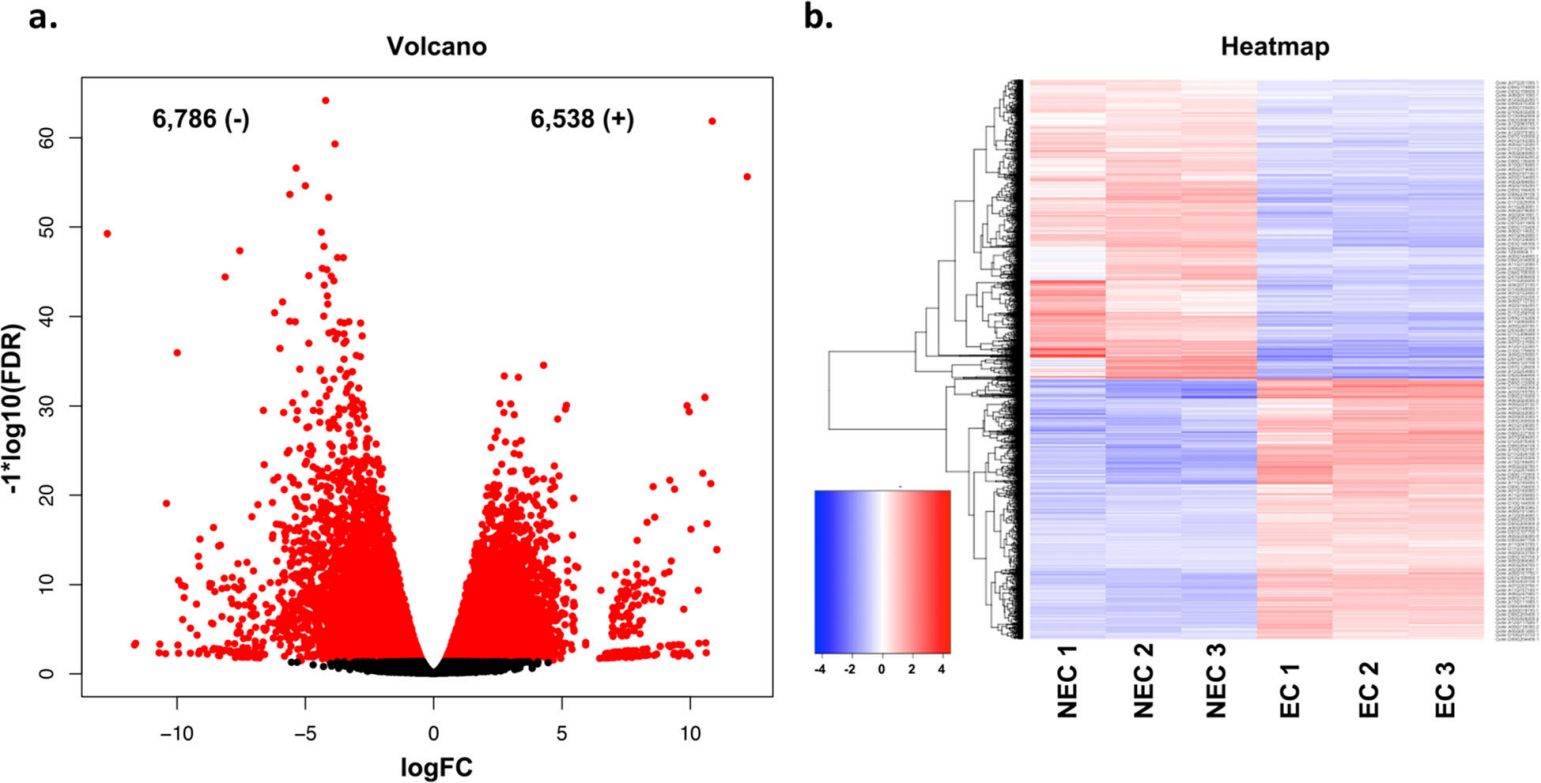
**a** Volcano plot of significant genes with FDR corrected *p*-values (<=.001). **b** Heatmap of gene expression profiles of NEC and EC callus cells

**Fig. 4:**
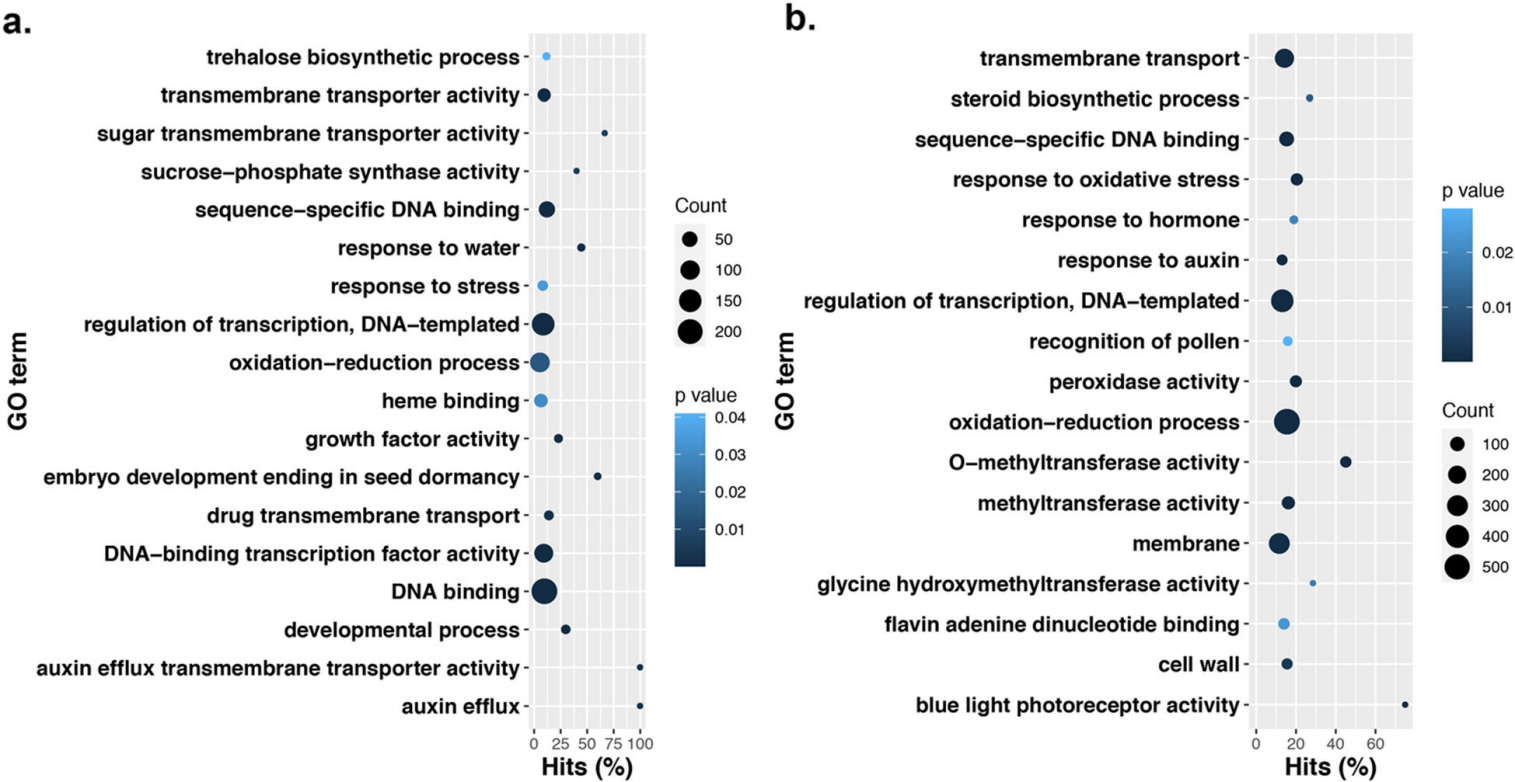
Gene ontology enrichment of **a** Significant upregulated genes (6786) and **b** Significantly downregulated genes in EC (6538)

For each callus cell type, an average of 36.8 and 42.7 million read pairs were produced for NEC and EC samples, respectively (BioProject ID PRJNA629328). A total of 39,105 genes were expressed in both NEC and EC callus cells, Fig. 2. Overall, a total of 5333 genes were differentially expressed with 2534 upregulated and 2799 downregulated in EC (Additional file 1: Table S1). Among these genes, a total of 144 were uniquely expressed in NEC cells, whereas, 174 genes were unique to EC (Fig. 2, Additional file 2: Table S2). Genes with unique expression to either EC or NEC cells were mostly genes of unknown function. Genes with annotations that were highly expressed in NEC include ROTUNDIFOLIA-LIKE 21, gibberellin-regulated, WRKY DNA binding, and others. Genes with high expression unique to EC callus with annotations include MYB family transcription factor, cytochrome C biogenesis, ROTUNDIFOLIA-LIKE 10, heat shock, and several others (Additional file 1: Table S1). Gene expression fold changes ranged from − 12 to 12 in EC when compared to NEC, (Additional file 1: Table S1). A slightly larger set of transcripts indicated transcriptional downregulation from NEC to EC, with a total of 6786 genes (logFC ≥1) becoming downregulated in EC; while 6538 (logFC ≥1) were upregulated (Fig. 3a and b, Additional file 1: Table S1). The top 10 genes with the sharpest fold changes in upregulation EC versus NEC is a gene with a nodulin late domain (*Gohir.D11G247300.1*), and 9 genes with unknown function (Table 2). The top 10 genes with the sharpest fold changes in downregulation are a gibberellin-regulate homolog (*Gohir.D04G048900.1*), 4 genes with unknown function, a gene with an S-adenosyl-l-methionine decarboxylase (AdoMetDC) leader peptide, a ROTUNDIFOLIA like gene (*Gohir.D07G179500.2*), and 60S ribosomal protein (Table 2). Gene ontology enrichment analysis of the upregulated genes in EC (Fig. 2a and b) discovered genes enriched in biological processes such as trehalose biosynthesis, transmembrane transporter, sugar transport, iron binding, growth factor activity, embryo development, development, auxin efflux, and response to stress among other processes related to cellular reprogramming and differentiation (Fig. 4a, Additional file 3: Table S3). The most pronounced enriched category was DNA binding and transcription factor activity with 309 genes combined, followed by regulation of transcription (155 genes), membrane (228 genes), oxidation reduction (105 genes), and transmembrane transport (80 genes) (Additional file 3: Table S3). Notable categories that were downregulated were categorized by assigned GO terms enriched for oxidation-reduction (524 genes), transmembrane transport (233 genes), response to hormones and auxin (48 genes), methyltransferase activity (112 genes), and peroxidase activity (58 genes) (Additional file 4: Table S4).

**Table 2:** Top 10 genes upregulated in EC relative to NEC

Gene	Control	Observed effect	logFC	PValue	FDR	Functional domains (descriptions)	Functional domains (domain name)	Athaliana_Defline	Ghirsutum_Defline
Gohir.D11G247300.1	NEC	EC	12.2179854	2.64E-60	2.44E-56	Nodulin_late	Nodulin_late		
Gohir.A09G068700.1	NEC	EC	11.0311639	2.26E-16	1.41E-14	DUF3963	DUF3963		
Gohir.A03G181200.1	NEC	EC	10.8541708	5.80E-67	1.34E-62	#N/A	#N/A		
Gohir.A05G291150.1	NEC	EC	10.7942425	2.50E-24	5.12E-22	DUF4912	DUF4912		
Gohir.A09G187300.1	NEC	EC	10.6509234	1.66E-19	1.72E-17	#N/A	#N/A		
Gohir.A11G045350.1	NEC	EC	10.6244362	0.00116229	0.00472295	#N/A	#N/A		
Gohir.D12G136400.1	NEC	EC	10.6014584	6.50E-05	0.0003752	#N/A	#N/A		
Gohir.A08G165050.1	NEC	EC	10.5716199	1.71E-34	1.16E-31	#N/A	#N/A		
Gohir.A07G162000.3	NEC	EC	10.488521	1.52E-25	3.68E-23	#N/A	#N/A		
Gohir.D11G067750.1	NEC	EC	10.3328265	7.12E-05	0.00040677	#N/A	#N/A		
Gohir.D04G048900.1	NEC	EC	−9.94544461036326	1.19E-12	3.79E-11	GASA	GASA	GASR7 - Gibberellin-regulated GASA/GAST/Snakin family protein precursor, expressed; GAST1 protein homolog 4	GAST1 protein homolog 4
Gohir.A09G040900.1	NEC	EC	−9.974189713011	0.0001288	0.0006863	#N/A	#N/A		
Gohir.D06G144900.2	NEC	EC	−9.98990989847144	1.08E-39	1.16E-36	#N/A	#N/A		
Gohir.D10G179800.1	NEC	EC	−10.4131140476177	5.93E-22	8.57E-20	VanY	VanY		
Gohir.D05G002150.1	NEC	EC	−10.4414002634445	0.00169419	0.00655082	#N/A	#N/A		
Gohir.A11G280900.1	NEC	EC	−10.675999492066	9.84E-05	0.00054239	Phage_holin_3_7	Phage_holin_3_7		
Gohir.D13G226251.1	NEC	EC	−10.6995332832501	0.00118551	0.00480505	#N/A	#N/A		
Gohir.D05G008300.1	NEC	EC	−11.6191438066039	7.96E-05	0.00044992	AdoMetDC_leader	AdoMetDC_leader	S-adenosyl-l-methionine decarboxylase leader peptide, putative, expressed; conserved peptide upstream open reading frame 9	conserved peptide upstream open reading frame 9
Gohir.D07G179500.2	NEC	EC	−11.6654565629349	0.00012965	0.00069003	DVL	DVL	expressed protein; ROTUNDIFOLIA like 21	ROTUNDIFOLIA like 21
Gohir.D08G229750.1	NEC	EC	−12.7143630581706	1.18E-53	5.45E-50	Ribosomal_L18A, DUF1891	Ribosomal_L18A, DUF1891	60S ribosomal protein L18a, putative, expressed; Ribosomal protein L18ae/LX family protein	Ribosomal protein L18ae/LX family protein

### Hierarchal clustering and GO enrichment of key clusters

**Fig. 5:**
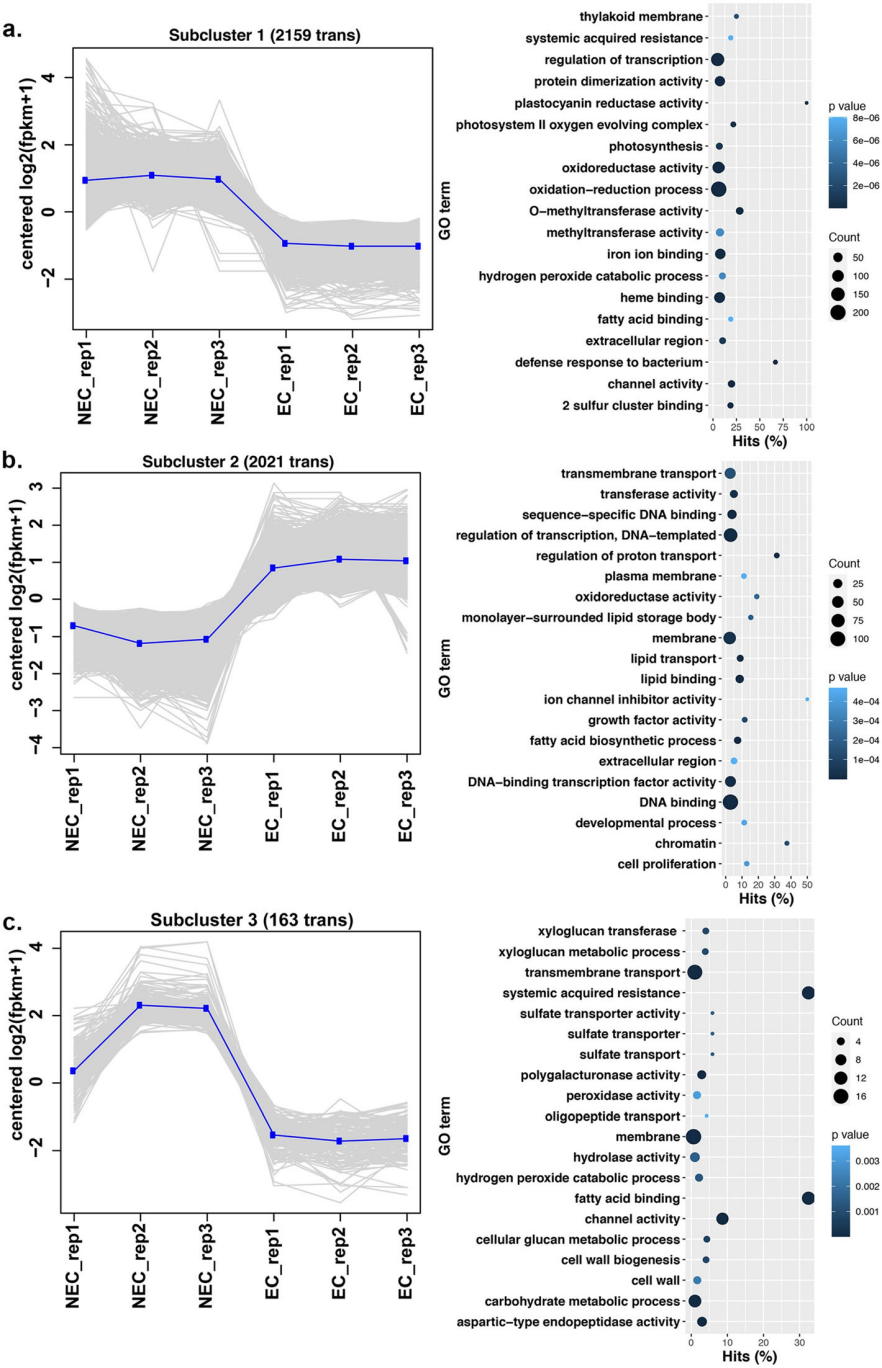
Hiarachal clustering and Gene Ontology enrichment of gene expression profiles. On the left, clustered genes whose expression profiles are at least 1 logFC and error-corrected *p*-value is ≤ .001. Genes in each subcluster were analyzed for gene ontology enrichment (right)

Hierarchal clustering of statistically significant gene expression profiles identified 3 distinct sub-clusters. (Fig. 5a-c, Additional file 5: Table S5). Subcluster 1 contained 2186 genes, and the expression trend of these genes ranged from moderate to low in NEC and EC callus, respectively (Fig. 5a, Additional file 5: Table S5). Processes enriched in this subcluster were assigned to oxidation reduction, methyltransferase activity, iron binding, and defense (Fig. 5a, Additional file 6: Table S6). Subcluster 2 contained 675 genes, and showed an upward trend in expression in EC callus and enrichment of processes related to transcription factor activity, membrane, growth factor, and developmental processes (Fig. 5b, Additional file 7: Table S7). Subcluster 3 contains 183 genes, and specifically showed a more discreet trend of higher expression in NEC and lower expression in EC callus, with enrichment in processes such as membrane and transport, peroxidase activity, carbohydrate metabolism, and fatty acid binding (Fig. 5c, Additional file 8: Table S8).

### Phytohormone gene expression profiles in NEC and EC callus

**Fig. 6:**
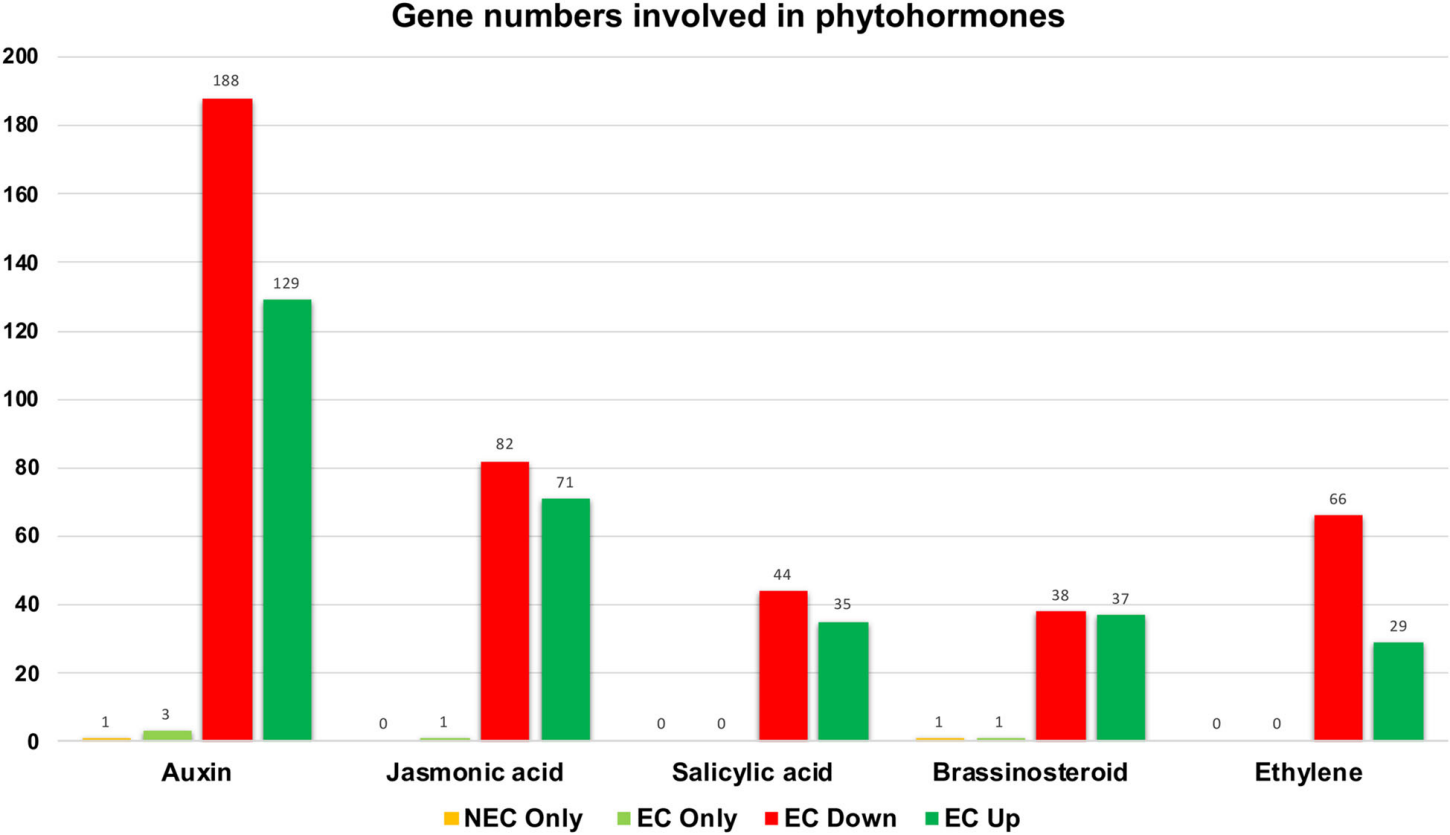
Genes involved in the synthesis of phytohormones

Genes involved in phytohormone signaling and biosynthesis are crucial for the transition from NEC to EC. In total, there are 6132 genes annotated as a phytohormone in the *Gossypium hirsutum* (CV TM1) reference genome assembly [49]. In general, we observed an abundance of phytohormone related genes being downregulated in EC callus relative to NEC (Table 3). Abscisic acid and auxin related genes were the categories with the most abundant differentially expressed genes, including 1508 and 1332 genes, respectively (Table 3). Expression magnitudes ranged from − 10 (logFC) in genes involved in gibberellic acid to 9 (logFC) in genes involved in abscisic acid (Additional file 9: Table S9 and Fig. 6). Genes annotated as cytokinin, gibberellic acid, jasmonic acid, salicyic acid, ethylene, and brassinosteroid were differentially expressed in NEC and EC (Table 3 and Additional file 9: Table S9).

**Table 3:** Phytohormone genes involved in NEC and EC callus

Phytohormone	No. Genes	No. Upregulated (> 1 FC)	No. Downregulated (> 1 FC)	logFC range
Auxin	1332	129	188	−8 to 7
Cytokinin	355	42	47	−9 to 5
Gibberellic acid	461	48	71	−10 to 8
Abscisic acid	1508	150	213	−8 to 9
Jasmonic acid	781	82	108	−8 to 5
Salicyic acid	593	74	88	−5 to 5
Ethylene	782	82	129	−6 to 5
Brassinosteroid	320	47	40	−8 to 8

### Transcription factors and signaling cascades

Transcription factors and cellular signaling genes that regulate reprogramming and differentiation during somatic embryogenesis were differentially expressed. We found 24 different classes of transcription factors that were significantly differentially expressed (Additional file 10: Table S10). The class with the most abundant genes was the GRAS (11 genes), followed by TGA like (9), MADS-box (9), CBF/NF-Y/archeal histone domain (9), GATA (8), and others with 7 or fewer members (Additional file 10: Table S10). Specific genes with known involvement in embryogenesis were individually analyzed for expression profiles in NEC and EC callus in Jin668. Our data shows that noteworthy embryogenesis transcription factors are lowly expressed in NEC callus with several key genes that are sharply upregulated in EC (Table 4). For example, the gene with the highest upregulation is *LEAFY COTYLEDON 1* (LEC1), with the dominant copy residing in the D-subgenome (Table 4). The next highly expressed embryogenesis related gene was the morphogenic regulator *BABY BOOM* (BBM) with the dominant copy on the A-subgenome, followed by *FUSCA* (FUS3) and *AGAMOUS-LIKE15* (AGL15) both with the dominant copies on the D-subgenome. Interestingly, *WUSHEL* (WUS) had very little to no expression with no apparent subgenome bias (Table 4).

**Table 4:** Embryogenesis genes and their expression values in Jin668

			A-Subgenome			D-Subgenome		
GeneID	Gene description	Arabidopsis Ortholog (GeneID)	GeneID	NEC TMM	EC TMM	GeneID	NEC TMM	EC TMM
*WOX5*	WUS-related homeobox 5	AT3G11260	Gohir.A10G233000	5.015	0.043	Gohir.D10G245300	2.261	0.234
*WUS*	*WUSHEL*	AT2G17950	Gohir.A10G098300	0.223	0.773	Gohir.D10G089500	0	0
			Gohir.A12G059800	0	0	Gohir.D12G060100	0	0
*WRKY2*	WRKY2	AT5G56270	Gohir.A08G026100	3.614	6.522	Gohir.D08G036600	5.855	6.839
			Gohir.A13G154100	3.503	6.941	Gohir.D13G158700	2.199	4.291
*GRD/RKD4*	GROUNDED	AT5G53040	Gohir.A03G051800	0.092	0.682	Gohir.D03G115300	0.027	0.351
*BBM*	BABY BOOM	AT5G17430	Gohir.A08G227000	4.889	26.808	Gohir.D08G247400	0.372	8.677
*LEC1*	LEAFY COTYLEDON1	AT1G21970	Gohir.A13G132600	4.363	85.081	Gohir.D13G136000	6.251	110.289
			Gohir.A08G025100	0.526	8.433	Gohir.D08G035600	2.069	15.03
*FUS3*	FUSCA	AT3G26790	Gohir.A07G230400	0.98	17.366	Gohir.D07G237600	1.353	23.266
*ABI3*	ABSCISIC ACID INSENSITIVE3	AT3G24650	Gohir.A07G154900	0.3	6.25	Gohir.D07G161100	0.047	1.09
*AGL15*	AGAMOUS-LIKE15	AT5G13790	Gohir.A08G141500	0.193	2.283	Gohir.D08G162600	0.485	4.502
			Gohir.A12G100400	0.948	10.919	Gohir.D12G103400	0.901	14.455

### Regulation of DNA methylation in NEC and EC

DNA methylation has a critical role in governing gene expression. In general, we observed a larger downregulation of genes regulating various methylation pathways in EC. We identified 226 genes with at least one-fold change in EC (71 upregulated, 155 downregulated) relative to NEC (Additional file 11: Table S11). Of the downregulated methylation genes, 15 proteins belong to protein family methyltransferase, 26 belong to plant invertase/pectin methylesterase inhibitors, 14 involved in S-adenosyl-L-methionine-dependent methyltransferase, 12 in the pectin methylesterase inhibitor family, and 35 in the O-methyltransferase superfamily (Additional file 11: Table S11). In contrast, very few methylation related protein families were upregulated in EC callus. Those that were include ribosomal protein L11 methyltransferase (3), tRNA methyltransferase (3), SAM dependent carboxyl methyltransferase (3), N6-adenine methyltransferase (3), and several others (Additional file 11: Table S11).

### RT-qPCR and validation of RNA-seq

**Fig. 7:**
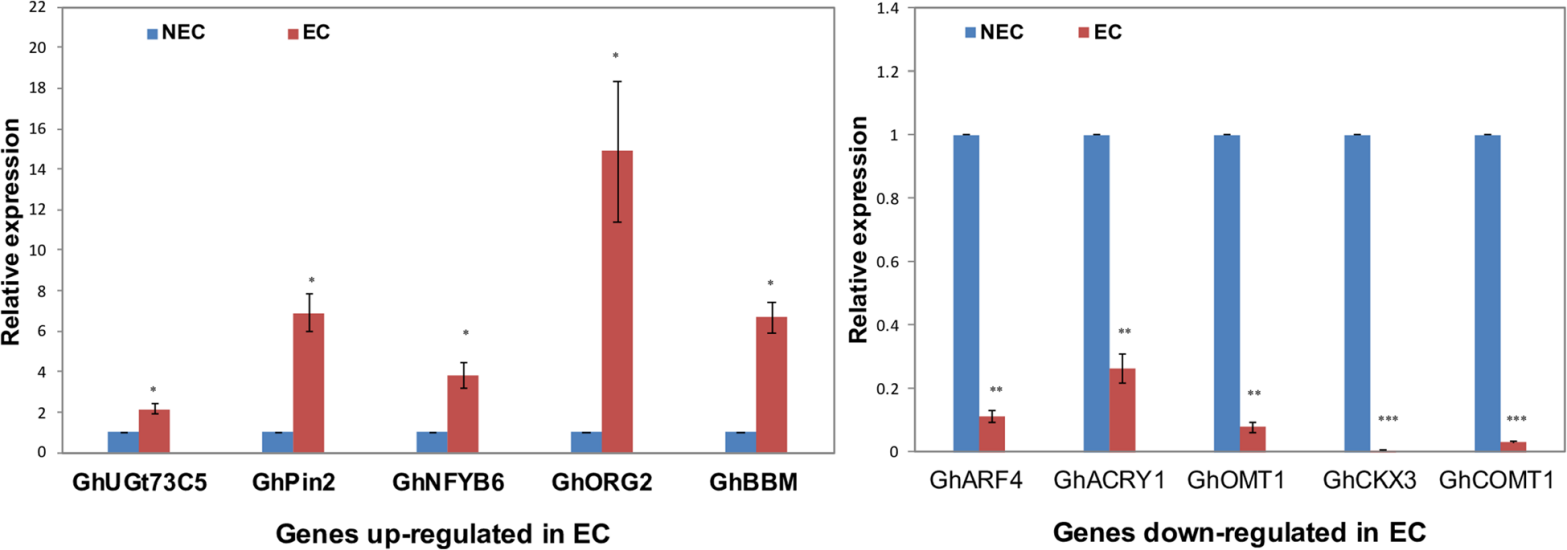
Evaluation of gene expression in embryogenic cells (EC) and non-embryogenic cells (NEC) by RT-qPCR analysis. Five of the selected genes were up-regulated (left); and the other five genes were down-regulated in embryogenic cells (right). *GhPP2A1* and *GhUb7* were used as the internal controls. Three biological replicates and three technical replicates were used for statistical analysis. Error bars indicate ±SE (*n* = 3). ΔΔCt method was used for qPCR analysis. Asterisks (*, ** or ***) indicate a significant difference between embryogenic cells (EC) and non-embryogenic cells (NEC) at *P* < 0.05, 0.01, or 0.001, respectively*,* by student’s *t*-test

To validate the RNA-seq data, ten critical embryogenesis related genes were selected for RT-qPCR analysis. All the primer pairs used in this test amplify a unique band with a unique melting curve peak (data not shown). The results showed (Fig. 7 and Additional file 12: Table S12) that five of the predicted genes were up-regulated in EC and are as follows: *GhUGt73C5*, UDP-glycosyltransferase 73C5-like gene (*Gohir.A01G078300.1*) [50]; *GhPin2*, auxin transporters, encodes an auxin efflux carrier (*Gohir.A05G001400.1*) [51]; *GhNFYB6*, nuclear factor Y subunit B-6, acts as a key component regulating embryogenesis and seed maturation in *Arabidopsis thaliana* (*Gohir.A05G176400.1*) [52]; *GhORG2*, transcription factor ORG2-like gene with basic helix-loop-helix (*Gohir.A09G106500.1*) [53]; *GhBBM*, AP2-like ethylene-responsive baby boom transcription factor (*Gohir.D08G247400.1*) [54]. The five deduced genes that were down-regulated in EC, i.e., *GhARF4*, auxin response factor 4 like, encodes a member of the ARF family of transcription factors (*Gohir.A05G074600.1*) [55]; *GhACRY1/BLU1*, cryptochrome-1-like, a flavin-type blue-light photoreceptor with ATP binding and autophosphorylation activity (*Gohir.A05G226100.1*) [56] (Pooam et al., 2018); *GhOMT1*, encoding a reticuline 7-O-methyltransferase-like gene (*Gohir.D02G181400.1*); *GhCOMT1*, raimondii caffeic acid 3-O-methyltransferase-like (*Gohir.D12G246900.1*) gene [57]; *GhCKX3*, cytokinin dehydrogenase 3 (*Gohir.D07G011600.1*) [58]. The results showed that the expression level evaluated by RT-qPCR exhibited similar patterns to the RNA-seq results of the ten genes, confirmed the results of RNA-seq. (Fig. 7, Additional file 12: Table S12).

## Discussion

Cotton is a major oilseed and fiber crop that could benefit from biotechnological improvement but is currently restricted to a few semi-recalcitrant genotypes that are amenable to low transformation frequencies and subsequent regeneration through somatic embryogenesis. Genotype specific recalcitrance to regeneration is common in most crop speciesand has proven to be a profound bottleneck in applying the latest advances in biotechnology to elite breeding material. In monocots, several advancements have been made in ectopically expressing key transcription factors (eg, *BBM/WUS2*) to improve or enhance the development of embryogenic cell formation [40]. However, this system does not directly translate to dicot system improvement. Here, we describe a gene expression changes during critical developmental time points in a non-recalcitrant genotype, Jin668 [31]. Our comparative results identified many genes of unknown function are differentially expressed between NEC to EC in Jin668. Of those with annotations, we found the expression of many transcription factors increased in EC cells, such as TF NikR, AP2/ERF, MYB-CC, subunit A of Y, PWRKY, BREVIS RADIX, GTE1/GTE6, CBF/NF-Y, GATA, GRAS, IIF, MYC/MYB, TGA, k-box, MADS-box, NFYB/HAP3, TCP, GT3 and members of the heat shock TF family. Transcription factors are known to have important roles that guide the regulation of plant differentiation and development [37]. For example, BREVIS RADIX and MYB are involved in cell proliferation and elongation, GATA TFs function in cell differentiation and early embryos initiation and development, TCP are involved in cell spatial orientation and determination, Both GRAS and k-box regulate plant reprogramming, and MYC/MYB, MYB-CC, MADS-box, CBF/NF-Y, AP2/ERF, GT3 and TGA transcription factors function in the response to phytohormones and/or biotic or abiotic stresses [59, 60]. We also identified several genes that were upregulated in EC callus that are involved in phytohormone response, such as genes involved in carrier, efflux, response to auxin, cytokinin dehydrogenase, and homeobox domain (Fig. 6). Plant cells use these TFs to transmit signals to respond to environmental stimuli or initiate developmental programs. Furthermore, studies have shown non-cell-autonomous transcription factors are crucial for successful somatic regeneration [61].

Cellular proliferation and elongation are also very important for callus development during somatic regeneration. In Jin668 at the EC stage, we found upregulation of the TFs BREVIS RADIX (BRX) and MYC/MYB when compared to the NEC stage. In a study by Mouchel et al., their research team demonstrated that the *BRX* gene controlled cell proliferation and elongation in the growth zone of root tips in Arabidopsis [62]. Thereafter, BRX was found to be involved in regulation of brassinosteroid biosynthesis and maintenance to keep brassinosteroid biosynthesis above a critical threshold, which ultimately affects lateral root development in Arabidopsis [63].

We also found upregulation of 12 *GATA* genes which indicates that they play an important role in embryo initiation in cotton. GATA TFs are widely distributed in fungi, animals, and plants consist of a protein family containing a DNA-binding domain recognizing the DNA consensus sequence (A/T) GATA(A/G) and one or two highly conserved type-IV zinc fingers (C-X2-C-X17–20-C-X2-C) [64]. In plants, GATA TF expression is involved in light-mediated processes such as flowering, maturation, petal differentiation and expansion, and embryo development [65]. In the *Gossypium* genus, 30 GATA TFs have been identified [64]. Moreover, Nawy et al. reported that a GATA transcription factor, HANABA TARANU (HAN) was required to position the inductive Arabidopsis pro-embryo boundary and revealed that HAN regulated transcription in the basal pro-embryo [66].

Previous studies suggest that GRAS transcription factors act as essential regulators, not only in polar development [67] and reprogramming [68], but also in response to biotic and abiotic stresses [69, 70], and auxin response [71, 72]. Stuurman et al. reported that *HAM* (mutant hairy meristem) is a gene encoding a putative transcription factor of the *GRAS* family and mediates the signal from differentiating cells. This mechanism controls signals from differentiating tissues that extrinsically control stem cell fate in the shoot apex [59]. We found 11 *GRAS* genes upregulated in EC, suggesting that the upregulation of these genes is associated with embryo initiation and formation during somatic regeneration in cotton.

Previous work has shown that transient activation or ectopic expression of one or several transcription factors can trigger the transition from NEC to EC or increase the frequency of EC formation. These include *WOX5* [73], *WUSHEL* [74], *WRKY2* [75], *GRD/RKD4* [76], *BBM* [40], *LEC1* [54], *FUS3* [77], *ABI3* [78], and *AGL15* [79]. For example, the AP2/ERF TF *BBM* in plants induced plentiful cell regeneration and was used to produce a large number of somatic embryos that could developed to seedlings [80]. Furthermore, ectopic expression of *BBM* derived from *Brassica napus* was transformed into pepper and obtained efficiently regenerated transgenic plants from recalcitrant sweet pepper (*C. annuum*) varieties [42]. Our data revealed dramatic upregulation of *GRD/RKD4, BBM, LEC1, FUS3, ABI3 and AGL15* genes, both in A- and D-Subgenome in EC during the somatic embryogenesis. We also found four genes annotated as *WRKY2* that were upregulated in EC with subtle fold changes (1.18 to 1.95 logFC). Interestingly, only one of the four copies of *WUS* (*Gohir.A10G0998300*), was found up-regulated in EC callus, while the other three genes were not expressed, suggesting this homeologous subgenome specific gene is essential for somatic embryogenesis in cotton. Moreover, unlike the gene mentioned above, two *WOX5* genes, *Gohir.A10G233000* and *Gohir.D10G245300*, were found down-regulated in EC callus (Table 4). We hypothesize that this WOX5 transcription factor may be critical in triggering the embryo initiation, while the other transcription factors contribute as regulatory or maintenance factors that are critical to embryo formation and development. Furthermore, this homeobox gene was reported to contribute to signaling spiral differentiation of stem cells in *Arabidopsis thaliana*; while loss of WOX5 function in the root meristem stem cell niche caused terminal differentiation in distal stem cells [81]. Our data also indicates that a series of related transcription regulators are involved in signaling of embryo initiation and formation in cotton.

Phytohormone sensing is critical to cell fate reprogramming during somatic embryogenesis. Our results showed that during conversion from NEC to EC, a series of genes involved in the biosynthesis and transportation of auxin and cytokinin are upregulated, which indicates that these two phytohormones play important roles during the initiation of the embryos.

Auxins are key components to regeneration, and their exogenous application has shown to recuperate the embryogenic potential of mitotically quiescent somatic cells [82]. In *Triticum aestivum*, regeneration efficiency was associated with auxin exposure time and catalase metabolism during somatic embryogenesis. Induction of pluripotent cells, termed, “callus,” by auxin represents a typical cell fate change required for plant in vitro regeneration [83]. Studies have shown that relative signaling through MONOPTEROS (MP)/ARF 5 was required for shoot formation in Arabidopsis callus. Moreover, variants of MP expression revealed that this gene can promote de novo shoot formation in tissues that are normally recalcitrant [84]. Cytokinin also plays a fundamental role in cell fate reprogramming and is implicated in the process of shoot organogenesis [85]. Cytokinin promotes shoot regeneration through the up-regulation of *WUS*, and recent studies demonstrated that key cytokinin signaling components, namely type-B ARABIDOPSIS RESPONSE REGULATORSs (ARRs), directly bind the *WUS* promoter and regulate transcription of this gene [86]. These studies also suggest that the transcriptional regulation of early embryo patterning to hormonal control of plant callus cell initiation is comparable to similar strategies for stem cell formation in the animal kingdom [86]. Furthermore, it was found that the *WUS* expression is controlled by the ratio of cytokinin with auxin [87]. Our results show approximately 3 times more auxin related genes being upregulated in EC than cytokinin genes (129 vs. 42), suggesting a critical role for these genes in somatic embryogenesis in cotton.

## Conclusions

Understanding endogenous changes in developmental biology at the molecular level is paramount to developing strategies toward genotype independent transformation and subsequent whole plant regeneration. Here we describe the transcriptional landscape of two critical developmental stages of somatic embryogenesis, NEC cells and EC cells in a highly regeneratable cotton genotype, Jin668. Our study identified genes in this non-recalcitrant genotype that are differentially expressed and categoriezed them according to various biological processes that are important in somatic embryogenesis, such as phytohormone signaling, transcription factors and signaling cascads, and regulation of DNA methylation. We identified a list of candidate genes that will be important to follow up functional studies that characterize their functional role in enabling embryo formation in cotton. Optimizing transformation and regeneration systems is a large stride toward realizing the promises of synthetic biology and genome editing. We anticipate that this work will lead to new understandings of somatic embryogenesis in upland cotton and other dicot species.

## Methods

### Genotype and plant material source, callus, and sampling

The formal identification of the plant material used in this study was performed by Dr. Shuangxia Jin of Huazhong Agricultural University. The non-recalcitrant plant genotype used in this study, Jin668 (*Gossypium hirsutum* L.), was developed through successive regeneration acclimation (SRA) and is described in detail in [88]. Jin668 seeds (voucher specimens) are available through Material Transfer Agreement (MTA) with Dr. Shuangxia Jin of Huazhong Agricultural University. Seeds were geminated in April of 2018 at the Clemson University Systems Genetics lab using the following procedure: Seeds were sacrificed by sulfuric acid for 1 min, then rinsed under running deionized water for 2 h. The seeds were washed with 70% ethanol for 1 min and then washed three times with sterile deionized water. The seeds then were sterilized by shaking them in a flask containing 100 mL of 10% commercial bleach (+ 2 drops of Tween-20) for 5 min and rinsed three times with sterile DW. Finally, they were cultured in Magenta boxes on germination media (4.33 g/L Murashige and Skoog (MS) salts (MS basal salts mixture; PhytoTechnology Laboratories, cat. no. M524), 2% glucose (PhytoTechnology Laboratories, USA), pH 6.0, 0.26% Phytagel (PhytoTechnology Laboratories, USA) at 28 °C, under kept in dark conditions for 7 days. The 7-day hypocotyls were cut to 0.5–1.0 cm segments and placed on callus induction media [89], with media changes every 4 weeks. The plates were cultivated in a growth chamber with controlled conditions of 28 ± 1 °C, 16 h (day)/8 h (night) photoperiod, with light provided by cool-white fluorescent lamps at an irradiation of 135 μmolm^− 2^ s^− 1^, and 50% relative humidity. “The regeneration rates were calculated by the number of explants producing embryogenic callus of 300–330 explants (hypocotyl segments) within the 1.5-month tissue culture process, with 3 replicates. Two developmental stages were selected for transcriptome profiling based on callus morphology, non-embryogenic cells and embryogenic cells (Fig. 1). The non-embryogenic cells were cells that lacked visual queues of organization or signs of polarization. Embryogenic cells showed visual signs of differentiation and were considered to be at globular-stage. In an effort to minimize gene expression background signals that may originate from different explants from different petri dishes, samples for each developmental stage were prepare as follows: Either non-embryogenic callus (callus that were completely NEC or had both NEC and EC) or embryogenic callus cells were harvested with a sterile scalpel from 20 explants separately and combined into a single tube. This process was repeated three times to collect 3 biological replicates.

### RNA isolation and RNA sequencing

Total RNA was isolated from Jin668 callus material at different stages using a modified guanidine thiocyanate method [90]. RNA-Seq libraries were constructed using the Illumina TruSeq Stranded RNA kit (San Diego CA, USA) following the manufacturer’s recommendations with three biological replicates per condition. Paired-end sequences were collected on an Illumina NovaSeq through a third-party vendor. Raw sequence files were trimmed of low quality bases and adapter sequences with the trimmomatic software package [91].

### Identification of differentially expressed genes and enrichment analysis

Preprocessed reads were aligned to the *Gossypium hirsutum* v2.0 reference assembly (Chen et al., 2020) with the Bowtie2 short read aligner [92], and alignment files were coordinate sorted and indexed with samtools v1.3.1 [93]. Raw counts, FPKM and TMM values were generated with RSEM [94]. Counts for each replicate were collected in tabular format and normalized according to biological replicates to remove sample-specific effects with EdgeR [95]. Relative pairwise changes in gene level expression were made using a generalized linear model (glm) and classic pairwise comparison methods with the EdgeR package [95]. Genes abounding a *p*-value of ≤0.01 were corrected by the false discovery rate (FDR) of 0.05 [96]. Gene level fold-change values were output in tabular format and candidate genes abounding assigned thresholds were clustered into functional pathways with the GOSeq software tool [97] and filtered by error corrected *P*-values ≤0.05. Hierarchal clustering was performed by manually subsetting genes whose logFC was ≥ 2 and FDR corrected *p*-values were ≤.001. A matrix of log transformed gene expression values were input into the fastcluster R software package and clusters identified by cutting the tree at 50% max height.

### Gene validation through RT-qPCR

Quantitative real-time PCR was performed to verify the RNA-Seq data [55]. Three biological replicates of total RNA of cotton callus for RNA-seq were used for RT-qPCR. Briefly, 0.5–1 μg of total RNAs were reverse-transcribed to first strand cDNAs using M-MuLV reverse transcriptase (New England Biolabs, GA, USA) and primed by d(T)25-VN following manufacturer’s instructions. RT-qPCR was conducted on an iCycler iQ system (Bio-Rad, Hercules, CA, USA) in 20 μl of PCR reaction solution (SsoAdvanced™ Universal SYBR® Green Supermix, Bio-Rad, USA) with 100 nM of each primer; the input amount of the first strand cDNA per reaction is around equivalent to 0.6–1.2 ng of total RNA. Four technical repeats were used for each of the three biological replicates. PCR was conducted with the following program: an initial denature at 95 °C for 60 s, followed by 40 cycles of 95 °C for 20 s, 62 °C for 20 s, and 72 °C for 20 s. Finally, a melting curve was performed from 55.0 °C to 95.0 °C at 0.5 °C increments. The ΔΔCt method was used for real-time PCR analysis. Two reference genes, *GhPP2A1* [98] and *GhUBQ7* [88] were used as endogenous controls. Relative expression level was calculated using the 2-ΔΔCt formula. All primer pairs except for the reference genes used for examining the expression levels of cotton endogenous genes were designed based on the Jin668 RNAseq sequences and listed in Additional file 12: Table S12.

## Supplementary Information

**Additional file 1.** Differential gene expression profiles of NEC vs. EC in Jin668. Differentially expressed genes with functional annotations. The logFC value is the observed result in EC.

**Additional file 2.** Genes uniquely expressed in either NEC or EC cells. Genes with unique expression profiles in embryogenic or nonembryogenic callus with functional annotation from the definition line of *Gossypium hirsutum v2.0* and *Arabidopsis thaliana*.

**Additional file 3.** Gene Ontology enrichment of genes upregulated in EC (>=1 fold change). Gene ontology classification results of genes that are upregulated at least 1FC in embryogenic callus when compared to nonembryogenic callus.

**Additional file 4.** Gene Ontology enrichment of genes downregulated EC (>=1 fold change). Gene ontology classification results of genes that are downregulated at least 1FC in embryogenic callus when compared to nonembryogenic callus.

**Additional file 5.** Centered log2(fpkm+1) gene expression clusters of NEC and EC callus. Gene expression profiles of quantile normalized FPKM values for genes that were clustered by k-means clustering in NEC and EC callus.

**Additional file 6.** Subcluster 1 GO enrichment. Gene ontology enrichment of subcluster 1 as determined by k-means clustering

**Additional file 7.** Subcluster 2 GO enrichment. Gene ontology enrichment of subcluster 2 as determined by k-means clustering

**Additional file 8.** Subcluster 3 GO enrichment. Gene ontology enrichment of subcluster 3 as determined by k-means clustering

**Additional file 9.** Phytohormone genes expressed in NEC and EC cells in Jin668. A list of all phytohormone annotated genes in the v2.0 of *Gossypium hirsutum* reference assembly with logFC values in EC.

**Additional file 10.** Transcription factors with differential expression profiles in NEC and EC callus. A list of all annotated transcription factors in the v2.0 of *Gossypium hirsutum* reference assembly with logFC values in EC.

**Additional file 11.** Genes involved in DNA methylation regulation in NEC and EC callus. A list of all annotated genes involved in methylation regulation in the v2.0 of *Gossypium hirsutum* reference assembly with logFC values in EC. Functional annotation from domain scan and *Arabidopsis thaliana* definition lines from the best hit.

**Additional file 12.** Gene expression evaluation by RT-qPCR. The expression pattern of all the selected genes in embryogenic cells (EC) vs. non-embryogenic cells (NEC) confirmed the RNAseq results.

## Data Availability

The transcriptomic datasets generated during the current study are available undert the BioProject accession number PRJNA629328 in NCBI (https://submit.ncbi.nlm.nih.gov/subs/sra/SUB7346009/overview), and in the supplementary information files**; Table S1.** Differential gene expression profile of NEC vs. EC in Jin668. The logFC value is the observed result in EC; **Table S2:** Genes uniquely expressed in either NEC or EC cells; **Table S3.** Gene Ontology enrichment of genes upregulated in EC (> = 1 fold change); **Table S4.** Gene Ontology enrichment of genes downregulated EC (> = 1 fold change); **Table S5.** Centered log2(fpkm+ 1) gene expression clusters of NEC and EC callus; **Table S6.** Subcluster 1 GO enrichment; **Table S7**. Subcluster 2 GO enriochment; **Table S8**. Subcluster 3 GO enrichment; **Table S9**. Phytohormone genes expressed in NEC and EC cells in Jin668; **Table S10**. Transcription factors with differential expression profiles in NEC and EC callus; **Table S11**. Genes involved in DNA methylation regulation in NEC and EC callus; **Table S12**. Selected genes and primer sequences for qPCR expression validation.

## Abbreviations

NEC: Non-embryogenic callus
EC: Embryogenic callus
DEG: Differentially expressed genes
RT-qPCR: Quantitative reverse transcription polymerase chain reaction
SRA: Successive regeneration acclimation
PGR: Plant growth regulator
LEC1: LEAFY COTYLEDON 1
BBM: BABY BOOM
TF: Transcription factor

## References

[1] Dunwell JM. Transgenic approaches to crop improvement. J Exp Bot 2000; 51 Spec No:487–496. 10.1093/jexbot/51.suppl_1.487 10938856

[2] Mccabe DE, Swain WF, Martinell BJ, Christou P (1988). Stable transformation of soybean (glycine-max) by particle-acceleration. Bio-Technol.

[3] Cho MJ, Yano H, Okamoto D, Kim HK, Jung HR, Newcomb K, Le VK, Yoo HS, Langham R, Buchanan BB, Lemaux PG (2004). Stable transformation of rice (Oryza sativa L.) via microprojectile bombardment of highly regenerative, green tissues derived from mature seed. Plant Cell Rep.

[4] Gordon-Kamm WJ, Spencer TM, Mangano ML, Adams TR, Daines RJ, Start WG, O'Brien JV, Chambers SA, Adams WR, Willetts NG, Rice TB, Mackey CJ, Krueger RW, Kausch AP, Lemaux PG (1990). Transformation of maize cells and regeneration of fertile transgenic plants. Plant Cell.

[5] Altpeter F, Springer NM, Bartley LE, Blechl AE, Brutnell TP, Citovsky V, Conrad LJ, Gelvin SB, Jackson DP, Kausch AP, Lemaux PG, Medford JI, Orozco-Cardenas ML, Tricoli DM, Van Eck J, Voytas DF, Walbot V, Wang K, Zhang ZJ, Stewart CN (2016). Advancing crop transformation in the era of genome editing. Plant Cell.

[6] Baker M (2012). Method of the year 2011. Nat Methods.

[7] Manghwar H, Lindsey K, Zhang X, Jin S (2019). CRISPR/Cas system: recent advances and future prospects for genome editing. Trends Plant Sci.

[8] Bernardo R. Prospective targeted recombination and genetic gains for quantitative traits in Maize. Plant Genome. 2017;10(2). 10.3835/plantgenome2016.11.0118 28724082

[9] Leelavathi S, Sunnichan VG, Kumria R, Vijaykanth GP, Bhatnagar RK, Reddy VS (2004). A simple and rapid agrobacterium-mediated transformation protocol for cotton (Gossypium hirsutum L.): embryogenic calli as a source to generate large numbers of transgenic plants. Plant Cell Rep.

[10] Nam J, Matthysse AG, Gelvin SB (1997). Differences in susceptibility of Arabidopsis ecotypes to crown gall disease may result from a deficiency in T-DNA integration. Plant Cell.

[11] Liu W, Parrott WA, Hildebrand DF, Collins GB, Williams EG (1990). Agrobacterium induced gall formation in bell pepper (Capsicum annuum L.) and formation of shoot-like structures expressing introduced genes. Plant Cell Rep.

[12] Lowe K, Wu E, Wang N, Hoerster G, Hastings C, Cho MJ, Scelonge C, Lenderts B, Chamberlin M, Cushatt J, Wang L, Ryan L, Khan T, Chow-Yiu J, Hua W, Yu M, Banh J, Bao Z, Brink K, Igo E, Rudrappa B, Shamseer PM, Bruce W, Newman L, Shen B, Zheng P, Bidney D, Falco C, Register J, Zhao ZY, Xu D, Jones T, Gordon-Kamm W (2016). Morphogenic regulators baby boom and Wuschel improve monocot transformation. Plant Cell.

[13] Mordhorst AP, Toonen MAJ, de Vries SC (1997). Plant embryogenesis. Crit Rev Plant Sci.

[14] Yang XY, Zhang XL (2010). Regulation of somatic embryogenesis in higher plants. Crit Rev Plant Sci.

[15] Carman JG (1990). Embryogenic cells in plant-tissue cultures - occurrence and behavior. In Vitro Cell Dev B.

[16] Schmidt EDL, Guzzo F, Toonen MAJ, de Vries SC (1997). A leucine-rich repeat containing receptor-like kinase marks somatic plant cells competent to form embryos. Development.

[17] Ikeuchi M, Shibata M, Rymen B, Iwase A, Bagman AM, Watt L, Coleman D, Favero DS, Takahashi T, Ahnert SE, Brady SM, Sugimoto K (2018). A gene regulatory network for cellular reprogramming in plant regeneration. Plant Cell Physiol.

[18] Garcia-Martin G, Manzanera JA, Gonzalez-Benito ME (2005). Effect of exogenous ABA on embryo maturation and quantification of endogenous levels of ABA and IAA in Quercus suber somatic embryos. Plant Cell Tiss Org.

[19] Vahdati K, Bayat S, Ebrahimzadeh H, Jariteh M, Mirmasoumi M (2008). Effect of exogenous ABA on somatic embryo maturation and germination in Persian walnut (Juglans regia L.). Plant Cell Tiss Org.

[20] Eapen S, George L (1990). Influence of phytohormones, carbohydrates, aminoacids, growth supplements and antibiotics on somatic embryogenesis and plant differentiation in finger millet. Plant Cell Tissue Organ Cult.

[21] Mashayekhi K, Neumann KH (2006). Effects of boron on somatic embryogenesis of Daucus carota. Plant Cell Tissue Organ Cult.

[22] Shekhawat GS, Mathur S, Batra A (2009). Role of phytohormones and nitrogen in somatic embryogenesis induction in cell culture derived from leaflets of Azadirachta indica. Biol Plant.

[23] Yu T-A, Yeh S-D, Yang J-S (2001). Effects of carbenicillin and cefotaxime on callus growth and somatic embryogenesis from adventitious roots of papaya. Bot Bull Acad Sin.

[24] Komatsuda T, Kaneko K, Oka S (1991). Genotype ✕ sucrose interactions for somatic embryogenesis in soybean. Crop Sci.

[25] McKently AH (1995). Effect of genotype on somatic embryogenesis from axes of mature peanut embryos. Plant Cell Tissue Organ Cult.

[26] Niskanen A-M, Lu J, Seitz S, Keinonen K, Von Weissenberg K, Pappinen A (2004). Effect of parent genotype on somatic embryogenesis in scots pine (Pinus sylvestris). Tree Physiol.

[27] Parrott WA, Williams EG, Hildebrand DF, Collins GB (1989). Effect of genotype on somatic embryogenesis from immature cotyledons of soybean. Plant Cell Tissue Organ Cult.

[28] Trolinder NL, Xhixian C (1989). Genotype specificity of the somatic embryogenesis response in cotton. Plant Cell Rep.

[29] Condic ML (2014). Totipotency: what it is and what it Isn’t. Stem Cells Dev.

[30] Chu Z, Chen J, Sun J, Dong Z, Yang X, Wang Y, Xu H, Zhang X, Chen F, Cui D (2017). De novo assembly and comparative analysis of the transcriptome of embryogenic callus formation in bread wheat (*Triticum aestivum* L.). BMC Plant Biol.

[31] Li JY, Wang MJ, Li YJ, Zhang QH, Lindsey K, Daniell H, Jin SX, Zhang XL (2019). Multi-omics analyses reveal epigenomics basis for cotton somatic embryogenesis through successive regeneration acclimation process. Plant Biotechnol J.

[32] Xu ZZ, Zhang CJ, Zhang XY, Liu CL, Wu ZX, Yang ZR, Zhou KH, Yang XJ, Li FG (2013). Transcriptome profiling reveals Auxin and Cytokinin regulating somatic embryogenesis in different sister lines of cotton cultivar CCRI24. J Integr Plant Biol.

[33] Florentin A, Damri M, Grafi G (2013). Stress induces plant somatic cells to acquire some features of stem cells accompanied by selective chromatin reorganization. Dev Dyn.

[34] Ikeuchi M, Sugimoto K, Iwase A (2013). Plant callus: mechanisms of induction and repression. Plant Cell.

[35] Grafi G, Barak S (2015). Stress induces cell dedifferentiation in plants. Biochim Biophys Acta.

[36] Mendez-Hernandez HA, Ledezma-Rodriguez M, Avilez-Montalvo RN, Juarez-Gomez YL, Skeete A, Avilez-Montalvo J, De-la-Pena C, Loyola-Vargas VM (2019). Signaling overview of plant somatic embryogenesis. Front Plant Sci.

[37] Gruel J, Deichmann J, Landrein B, Hitchcock T, Jonsson H (2018). The interaction of transcription factors controls the spatial layout of plant aerial stem cell niches. NPJ Syst Biol Appl.

[38] Lotan T, Ohto M, Yee KM, West MAL, Lo R, Kwong RW, Yamagishi K, Fischer RL, Goldberg RB, Harada JJ (1998). Arabidopsis LEAFY COTYLEDON1 is sufficient to induce embryo development in vegetative cells. Cell.

[39] Zuo J, Niu QW, Frugis G, Chua NH (2002). The WUSCHEL gene promotes vegetative-to-embryonic transition in Arabidopsis. Plant J.

[40] Boutilier K, Offringa R, Sharma VK, Kieft H, Ouellet T, Zhang LM, Hattori J, Liu CM, van Lammeren AAM, Miki BLA, Custers JBM, Campagne MMV (2002). Ectopic expression of BABY BOOM triggers a conversion from vegetative to embryonic growth. Plant Cell.

[41] Srinivasan C, Liu Z, Heidmann I, Supena ED, Fukuoka H, Joosen R, Lambalk J, Angenent G, Scorza R, Custers JB, Boutilier K (2007). Heterologous expression of the BABY BOOM AP2/ERF transcription factor enhances the regeneration capacity of tobacco (Nicotiana tabacum L.). Planta.

[42] Heidmann I, de Lange B, Lambalk J, Angenent GC, Boutilier K (2011). Efficient sweet pepper transformation mediated by the BABY BOOM transcription factor. Plant Cell Rep.

[43] Florez SL, Erwin RL, Maximova SN, Guiltinan MJ, Curtis WR (2015). Enhanced somatic embryogenesis in Theobroma cacao using the homologous BABY BOOM transcription factor. BMC Plant Biol..

[44] Mitten DH (1985). Somatic embryogenesis in *Gossypium hirsutum* L.

[45] Davidonis GH, Hamilton RH (1983). Plant-regeneration from callus-tissue of Gossypium-Hirsutum-L. Plant Sci Lett.

[46] Trolinder NL, Goodin JR (1988). Somatic embryogenesis in cotton (Gossypium) .1. Effects of source of explant and hormone regime. Plant Cell Tiss Org.

[47] Gawel NJ, Robacker CD (1990). Genetic-control of somatic embryogenesis in cotton petiole callus-cultures. Euphytica.

[48] Lambe P, Mutambel HSN, Fouche JG, Deltour R, Foidart JM, Gaspar T (1997). DNA methylation as a key process in regulation of organogenic totipotency and plant neoplastic progression?. In Vitro Cell Dev-Pl.

[49] Chen ZJ, Sreedasyam A, Ando A, Song Q, De Santiago LM, Hulse-Kemp AM, Ding M, Ye W, Kirkbride RC, Jenkins J, Plott C, Lovell J, Lin YM, Vaughn R, Liu B, Simpson S, Scheffler BE, Wen L, Saski CA, Grover CE, Hu G, Conover JL, Carlson JW, Shu S, Boston LB, Williams M, Peterson DG, McGee K, Jones DC, Wendel JF, Stelly DM, Grimwood J, Schmutz J (2020). Genomic diversifications of five Gossypium allopolyploid species and their impact on cotton improvement. Nat Genet..

[50] Shin S, Torres-Acosta JA, Heinen SJ, McCormick S, Lemmens M, Paris MP, Berthiller F, Adam G, Muehlbauer GJ (2012). Transgenic Arabidopsis thaliana expressing a barley UDP-glucosyltransferase exhibit resistance to the mycotoxin deoxynivalenol. J Exp Bot.

[51] Wang MY, Zhao PM, Cheng HQ, Han LB, Wu XM, Gao P, Wang HY, Yang CL, Zhong NQ, Zuo JR, Xia GX (2013). The cotton transcription factor TCP14 functions in auxin-mediated epidermal cell differentiation and elongation. Plant Physiol.

[52] Kim HU, Jung SJ, Lee KR, Kim EH, Lee SM, Roh KH, Kim JB (2014). Ectopic overexpression of castor bean LEAFY COTYLEDON2 (LEC2) in Arabidopsis triggers the expression of genes that encode regulators of seed maturation and oil body proteins in vegetative tissues. FEBS Open Bio.

[53] Bailey PC, Martin C, Toledo-Ortiz G, Quail PH, Huq E, Heim MA, Jakoby M, Werber M, Weisshaar B (2003). Update on the basic helix-loop-helix transcription factor gene family in Arabidopsis thaliana. Plant Cell.

[54] Horstman A, Li M, Heidmann I, Weemen M, Chen B, Muino JM, Angenent GC, Boutilier K (2017). The BABY BOOM transcription factor activates the LEC1-ABI3-FUS3-LEC2 network to induce somatic embryogenesis. Plant Physiol.

[55] Li J, Tao X, Li L, Mao L, Luo Z, Khan ZU, Ying T (2016). Comprehensive RNA-Seq analysis on the regulation of tomato ripening by exogenous Auxin. PLoS One.

[56] Pooam M, Arthaut LD, Burdick D, Link J, Martino CF, Ahmad M (2018). Magnetic sensitivity mediated by the Arabidopsis blue-light receptor cryptochrome occurs during flavin reoxidation in the dark. Planta..

[57] Ho-Yue-Kuang S, Alvarado C, Antelme S, Bouchet B, Cezard L, Le Bris P, Legee F, Maia-Grondard A, Yoshinaga A, Saulnier L, Guillon F, Sibout R, Lapierre C, Chateigner-Boutin AL (2016). Mutation in Brachypodium caffeic acid O-methyltransferase 6 alters stem and grain lignins and improves straw saccharification without deteriorating grain quality. J Exp Bot.

[58] Gigli-Bisceglia N, Engelsdorf T, Strnad M, Vaahtera L, Khan GA, Yamoune A, Alipanah L, Novak O, Persson S, Hejatko J, Hamann T. Cell wall integrity modulates *Arabidopsis thaliana* cell cycle gene expression in a cytokinin- and nitrate reductase-dependent manner. Development. 2018;145(19):2049–77. 10.1242/dev.166678 30190280

[59] Stuurman J, Jaggi F, Kuhlemeier C (2002). Shoot meristem maintenance is controlled by a GRAS-gene mediated signal from differentiating cells. Genes Dev.

[60] Aguilar-Martinez JA, Sinha N (2013). Analysis of the role of Arabidopsis class I TCP genes AtTCP7, AtTCP8, AtTCP22, and AtTCP23 in leaf development. Front Plant Sci.

[61] Han X, Kumar D, Chen H, Wu S, Kim JY (2014). Transcription factor-mediated cell-to-cell signalling in plants. J Exp Bot.

[62] Mouchel CF, Briggs GC, Hardtke CS (2004). Natural genetic variation in Arabidopsis identifies BREVIS RADIX, a novel regulator of cell proliferation and elongation in the root. Genes Dev.

[63] Li J, Mo X, Wang J, Chen N, Fan H, Dai C, Wu P (2009). BREVIS RADIX is involved in cytokinin-mediated inhibition of lateral root initiation in Arabidopsis. Planta.

[64] Zhang Z, Zou X, Huang Z, Fan S, Qun G, Liu A, Gong J, Li J, Gong W, Shi Y, Fan L, Zhang Z, Liu R, Jiang X, Lei K, Shang H, Xu A, Yuan Y (2019). Genome-wide identification and analysis of the evolution and expression patterns of the GATA transcription factors in three species of Gossypium genus. Gene.

[65] Behringer C, Schwechheimer C (2015). B-GATA transcription factors - insights into their structure, regulation, and role in plant development. Front Plant Sci.

[66] Nawy T, Bayer M, Mravec J, Friml J, Birnbaum KD, Lukowitz W (2010). The GATA factor HANABA TARANU is required to position the proembryo boundary in the early Arabidopsis embryo. Dev Cell.

[67] Grimplet J, Agudelo-Romero P, Teixeira RT, Martinez-Zapater JM, Fortes AM (2016). Structural and functional analysis of the GRAS gene family in grapevine indicates a role of GRAS proteins in the control of development and stress responses. Front Plant Sci.

[68] Gallagher KL, Benfey PN (2009). Both the conserved GRAS domain and nuclear localization are required for SHORT-ROOT movement. Plant J.

[69] D'Alessandro S, Ksas B, Havaux M (2018). Decoding beta-cyclocitral-mediated retrograde signaling reveals the role of a detoxification response in plant tolerance to photooxidative stress. Plant Cell.

[70] Zhou S, Hu Z, Li F, Yu X, Naeem M, Zhang Y, Chen G (2018). Manipulation of plant architecture and fl owering time by down-regulation of the GRAS transcription factor SlGRAS26 in Solanum lycopersicum. Plant Sci.

[71] Qiao L, Zhang W, Li X, Zhang L, Zhang X, Li X, Guo H, Ren Y, Zheng J, Chang Z (2018). Characterization and expression patterns of Auxin response factors in wheat. Front Plant Sci.

[72] Hakoshima T (2018). Structural basis of the specific interactions of GRAS family proteins. FEBS Lett.

[73] Richards S, Wink RH, Simon R (2015). Mathematical modelling of WOX5- and CLE40-mediated columella stem cell homeostasis in Arabidopsis. J Exp Bot.

[74] Shinohara H, Matsubayashi Y (2015). Reevaluation of the CLV3-receptor interaction in the shoot apical meristem: dissection of the CLV3 signaling pathway from a direct ligand-binding point of view. Plant J.

[75] Guan Y, Meng X, Khanna R, LaMontagne E, Liu Y, Zhang S (2014). Phosphorylation of a WRKY transcription factor by MAPKs is required for pollen development and function in Arabidopsis. PLoS Genet.

[76] Jeong S, Eilbert E, Bolbol A, Lukowitz W (2016). Going mainstream: how is the body axis of plants first initiated in the embryo?. Dev Biol.

[77] Roscoe TJ, Vaissayre V, Paszkiewicz G, Clavijo F, Kelemen Z, Michaud C, Lepiniec LC, Dubreucq B, Zhou DX, Devic M (2019). Regulation of FUSCA3 expression during seed development in Arabidopsis. Plant Cell Physiol.

[78] Yotsui I, Saruhashi M, Kawato T, Taji T, Hayashi T, Quatrano RS, Sakata Y (2013). ABSCISIC ACID INSENSITIVE3 regulates abscisic acid-responsive gene expression with the nuclear factor Y complex through the ACTT-core element in Physcomitrella patens. New Phytol.

[79] Perry SE, Zheng Q, Zheng Y (2016). Transcriptome analysis indicates that GmAGAMOUS-like 15 may enhance somatic embryogenesis by promoting a dedifferentiated state. Plant Signal Behav.

[80] Lowe K, La Rota M, Hoerster G, Hastings C, Wang N, Chamberlin M, Wu E, Jones T, Gordon-Kamm W (2018). Rapid genotype "independent" Zea mays L. (maize) transformation via direct somatic embryogenesis. In Vitro Cell Dev Biol Plant.

[81] Sarkar AK, Luijten M, Miyashima S, Lenhard M, Hashimoto T, Nakajima K, Scheres B, Heidstra R, Laux T (2007). Conserved factors regulate signalling in Arabidopsis thaliana shoot and root stem cell organizers. Nature.

[82] Singla B, Tyagi AK, Khurana JP, Khurana P (2007). Analysis of expression profile of selected genes expressed during auxin-induced somatic embryogenesis in leaf base system of wheat (Triticum aestivum) and their possible interactions. Plant Mol Biol.

[83] Xu C, Cao H, Zhang Q, Wang H, Xin W, Xu E, Zhang S, Yu R, Yu D, Hu Y (2018). Control of auxin-induced callus formation by bZIP59-LBD complex in Arabidopsis regeneration. Nat Plants.

[84] Ckurshumova W, Berleth T (2015). Overcoming recalcitrance - Auxin response factor functions in plant regeneration. Plant Signal Behav.

[85] Hill K, Schaller GE. Enhancing plant regeneration in tissue culture: a molecular approach through manipulation of cytokinin sensitivity. Plant Signal Behav. 2013;8(10). 10.4161/psb25709. 10.4161/psb.25709 PMC4091070 23887495

[86] Zhang Z, Tucker E, Hermann M, Laux T (2017). A molecular framework for the embryonic initiation of shoot meristem stem cells. Dev Cell.

[87] Cheng ZJ, Zhu SS, Gao XQ, Zhang XS (2010). Cytokinin and auxin regulates WUS induction and inflorescence regeneration in vitro in Arabidopsis. Plant Cell Rep.

[88] Li J, Wang M, Li Y, Zhang Q, Lindsey K, Daniell H, Jin S, Zhang X (2018). Multi-omics analyses reveal epigenomics basis for cotton somatic embryogenesis through successive regeneration acclimation process. Plant Biotechnol J.

[89] Jin SX, Zhang XL, Liang SG, Nie YC, Guo XP, Huang C (2005). Factors affecting transformation efficiency of embryogenic callus of upland cotton (Gossypium hirsutum) with agrobacterium tumefaciens. Plant Cell Tiss Org.

[90] Suzuki Y, Mae T, Makino A (2008). RNA extraction from various recalcitrant plant tissues with a cethyltrimethylammonium bromide-containing buffer followed by an acid guanidium thiocyanate-phenol-chloroform treatment. Biosci Biotechnol Biochem.

[91] Bolger AM, Lohse M, Usadel B (2014). Trimmomatic: a flexible trimmer for Illumina sequence data. Bioinformatics.

[92] Langmead B, Trapnell C, Pop M, Salzberg SL (2009). Ultrafast and memory-efficient alignment of short DNA sequences to the human genome. Genome Biol.

[93] Li H, Handsaker B, Wysoker A, Fennell T, Ruan J, Homer N, Marth G, Abecasis G, Durbin R, Genome Project Data Processing S (2009). The sequence alignment/map format and SAMtools. Bioinformatics.

[94] Li B, Dewey CN (2011). RSEM: accurate transcript quantification from RNA-Seq data with or without a reference genome. BMC Bioinformatics.

[95] Robinson MD, McCarthy DJ, Smyth GK (2010). edgeR: a bioconductor package for differential expression analysis of digital gene expression data. Bioinformatics.

[96] Benjamini Y, Hochberg Y (1995). Controlling the false discovery rate - a practical and powerful approach to multiple testing. J R Stat Soc B Met.

[97] Young MD, Wakefield MJ, Smyth GK, Oshlack A (2010). Gene ontology analysis for RNA-seq: accounting for selection bias. Genome Biol.

[98] Artico S, Nardeli SM, Brilhante O, Grossi-de-Sa MF, Alves-Ferreira M (2010). Identification and evaluation of new reference genes in Gossypium hirsutum for accurate normalization of real-time quantitative RT-PCR data. BMC Plant Biol.

